# Low Protein Z Level: A Thrombophilic Risk Biomarker for Acute Coronary Syndrome

**DOI:** 10.1007/s12288-018-01073-7

**Published:** 2019-01-18

**Authors:** Artur Słomka, Ewa Żekanowska

**Affiliations:** Department of Pathophysiology, Nicolaus Copernicus University in Toruń, Ludwik Rydygier Collegium Medicum in Bydgoszcz, 9 Skłodowskiej-Curie Str., 85-094 Bydgoszcz, Poland

Dear Editor,

We found the paper penned by Ghozlan et al. [1] which was published in the *Indian Journal of Hematology and Blood Transfusion* profoundly interesting. The article is devoted to the measurement of the concentration of protein Z (PZ) in patients during the stable phase of acute coronary syndrome (ACS). PZ is a glycoprotein synthesized in the liver and in the vascular endothelium. Its main function is to inhibit, with the participation of another glycoprotein—ZPI (protein Z-dependent protease inhibitor), the active factor X [2]. The meta-analysis by Sofi et al. [3] proved that the low plasma concentration of PZ is an independent risk factor for the development of thrombosis.

The main observation made by Ghozlan et al. [1] is finding that ACS patients have a statistically significant lower concentration of PZ in their blood compared to the healthy individuals. A few methodological remarks come to mind when reading this publication. First of all, the authors used a number of criteria for the inclusion and exclusion of patients from the study; however, one of them, i.e. the use of oral anticoagulants as an exclusion criterion, raises the question of how patients who had suffered myocardial infarction were treated? Second of all, when interpreting the obtained results, it would be now valuable if the authors had presented the number of myocardial infarctions suffered by the patients and the time that passed between the myocardial infarction and the moment of taking a blood sample. Additionally, the authors should have described the control group in more detail. This seems particularly important in the context of age, considering the fact that a very strong negative correlation between age and PZ concentration in the experimental group was observed. Was a similar analysis carried out in the control group? A valuable observation made by the authors is the finding of a lower concentration of PZ in patients diagnosed with arterial hypertension. Nevertheless, this requires confirmation in subsequent studies due to the fact that other research has showed no difference in PZ concentration depending on the absence or presence of arterial hypertension in patients with coronary artery disease [4].

In the light of other papers, it would also be interesting to broaden the panel of tests to determine the concentration of ZPI and anti-PZ autoantibodies class IgG and IgM. The presence of circulating antibodies is relatively rare [5], but it would be interesting to determine whether the lower PZ concentration in the blood of ACS patients is due to the presence of these antibodies or not. The data presented in the work of Ghozlan et al. [1] are nevertheless compelling and constitute the basis for further pursuits of the diagnostic value of PZ in cardiovascular diseases.

## References

[1] Ghozlan MF, Mohamed AAEH, Eissa DS, Eldawy HS (2018). Low protein Z level: a thrombophilic risk biomarker for acute coronary syndrome. Indian J Hematol Blood Transfus.

[2] Vasse M (2011). The protein Z/protein Z-dependent protease inhibitor complex. Systemic or local control of coagulation?. Hamostaseologie.

[3] Sofi F, Cesari F, Abbate R, Gensini GF, Broze G, Fedi S (2010). A meta-analysis of potential risks of low levels of protein Z for diseases related to vascular thrombosis. Thromb Haemost.

[4] Słomka A, Piekuś A, Kowalewski M, Pawliszak W, Anisimowicz L, Żekanowska E (2018). Assessment of the procoagulant activity of microparticles and the protein Z system in patients undergoing off-pump coronary artery bypass surgery. Angiology.

[5] Pardos-Gea J, Ordi-Ros J, Serrano S, Balada E, Nicolau I, Vilardell M (2008). Protein Z levels and anti-protein Z antibodies in patients with arterial and venous thrombosis. Thromb Res.

